# Supplementary material for: Tag-seq: a convenient and scalable method for genome-wide specificity assessment of CRISPR/Cas nucleases
Source: Commun Biol. 2021 Jul 2;4:830. doi: 10.1038/s42003-021-02351-3 (PMC8253812; doi:10.1038/s42003-021-02351-3)
Supplement: Supplementary file 2 — Supplementary Information [file 42003_2021_2351_MOESM2_ESM.pdf]

Supplementary Figure 1

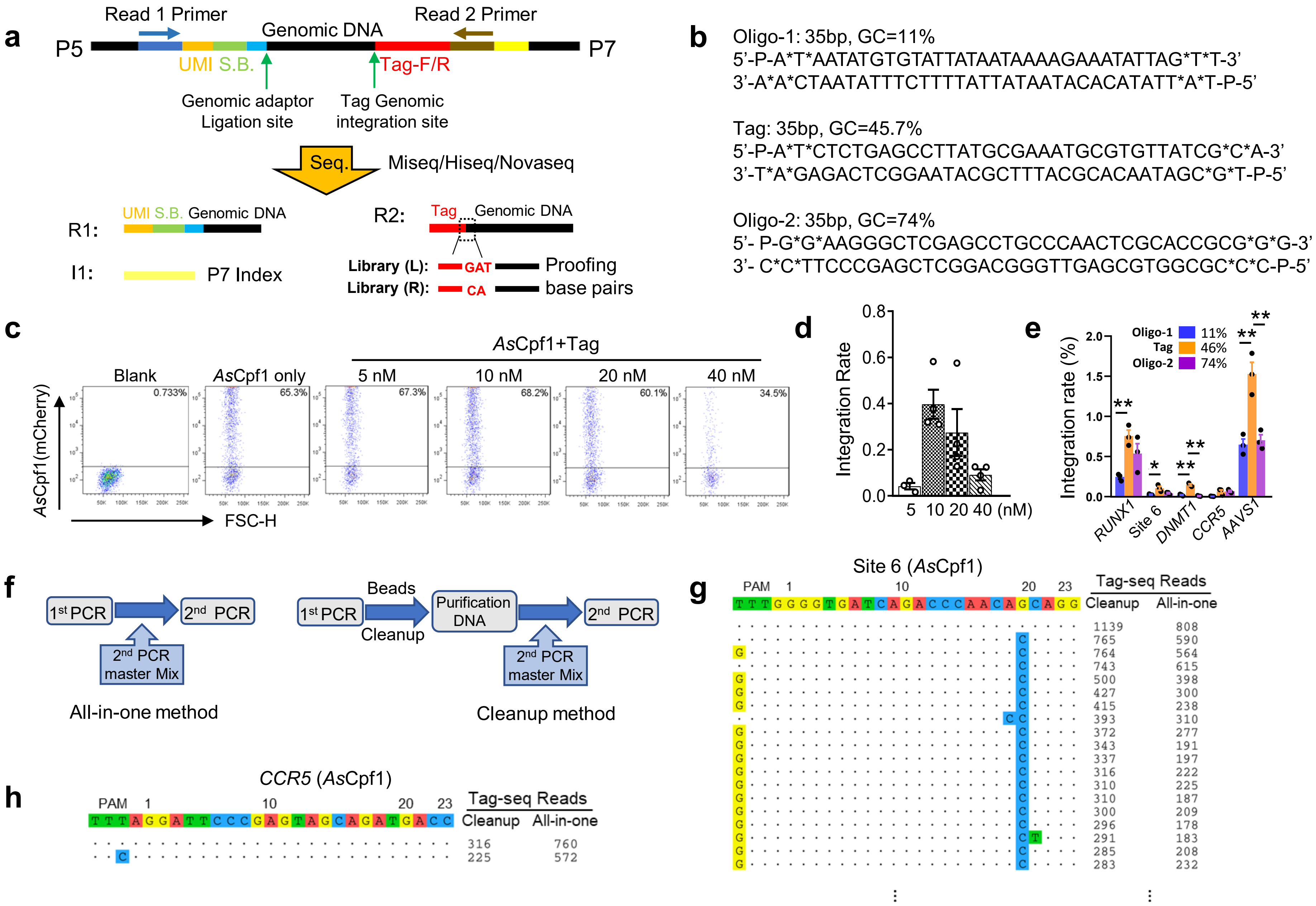

Supplementary Figure 1. The design and test of the Tag-seq.

Supplementary Figure 1. The design and test of the Tag-seq. (a) Schematic of the Tag-seq library. Paired-end sequencing was performed using a commercial platform such as Miseq, HiSeq or NovaSeq. All the information required to identify DSB sites, including the unique molecular index (UMI), sample barcode (S.B.), adaptor-genome ligation site, and Tag-genome integration site, can be retrieved directly from the paired R1/R2 reads. The "GAT" and "CA" nucleotide motifs in R2 were used for mispriming proof to increase specificity. P5/P7, flow cell binding sites containing P5/P7 sequences. R1/R2, Read1/Read2. I1, Index1 files. **(b)** The donor template DNA sequences. P, phosphorylation. *, phosphorothioate linkage. **(c)** Effects of the Tag concentration on cell transfection efficiency by PEI-based method. The 10 nM group showed comparable transfection efficiency to the Tag-untransfected control. **(d)** Examining the integration rate of the Tag at 5, 10, 20 and 40 nM concentrations with semi-quantitative PCR at 4 different genomic sites (also see Supplementary Fig. 2). **(e)** Deep-seq analyses of the integration rate among the oligo-1(GC content, ~11%), Tag (GC content, ~45.7%) and oligo-2 (GC content, ~74%) at *RUNX1*, Site 6, *DNMT1*, *CCR5* loci induced by *As*Cpf1 and *AAVS1 locus* induced by *Sp*Cas9. Mean values are presented with S.E.M, n=3 independent biological replicates. Student's t-test (Tag group versus oligo-1/oligo-2), **P* <0.05, ***P* <0.01. **(f)** Schematic of the all-in-one nested PCR procedure. **(g, h)** The all-in-one and cleanup PCR procedures led to comparable results at Site 6 and *CCR5* loci induced by *As*Cpf1. Due to the limited space, the off-targets of Site 6 were not completely displayed, and a full list was shown in Supplementary Fig. 3.

Supplementary Figure 2

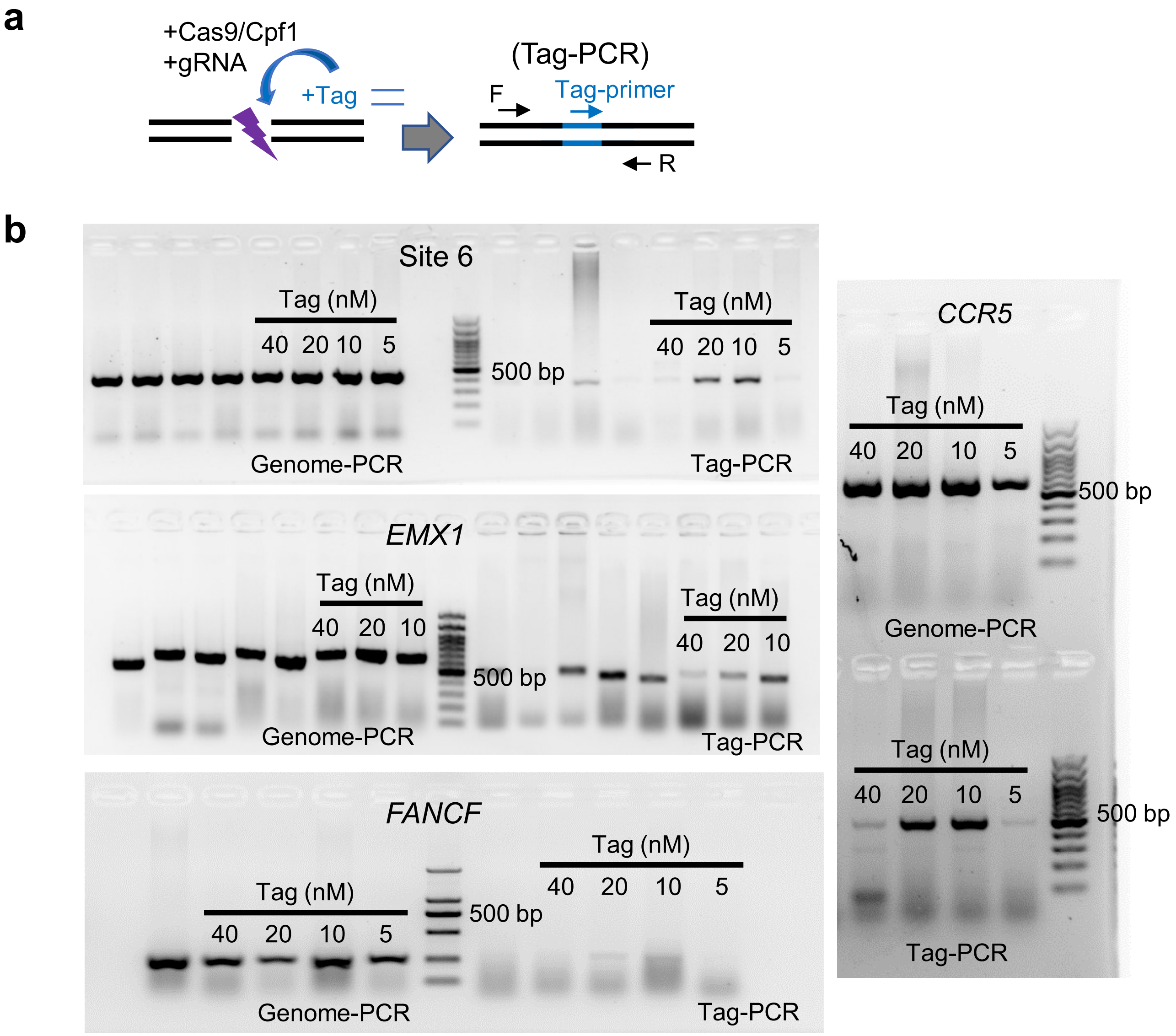

Supplementary Figure 2. Roughly examining the integration rate of the Tag with semi-quantitative PCR.

Supplementary Figure 2. Roughly examining the integration rate of the Tag with semi-quantitative PCR. (a) Schematic of the Genome-PCR and the Tag-PCR methods. Genome-PCR was the use of the gene specific F and R primers; Tag-PCR was the use of the Tag specific primer and a gene corresponding R primers. F/R, the gene specific forward/reversed primer. Tag-primer, the Tag specific primer. **(b)** The PCR results for the Genome-PCR and the Tag-PCR with 4 tested sites, Site6, *EMX1*, *FANCF*, and *CCR5*. The Integration Rate = (the Gray levels of the PCR produce using Tag-primer and R primers) / (the Gray levels of the PCR produce using gene specific F and R primers).

Supplementary Figure 3

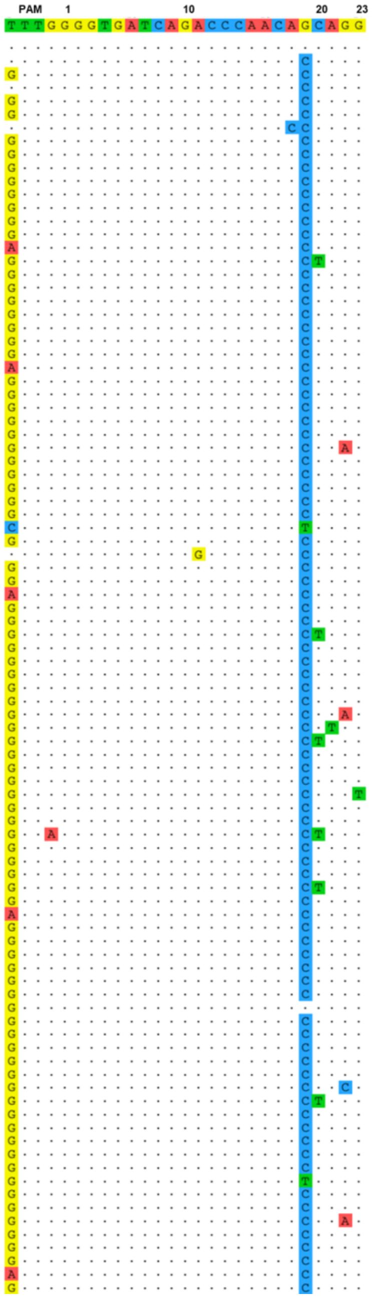

293T						MCF7
Cleanup			All in one			
3G	2G	1G	3G	2G	1G	3G
2650	1948	1139	1801	1308	808	1658
1861	1386	743	1341	966	615	857
1844	1357	764	1329	989	564	93
1718	1237	765	1349	1000	590	649
1158	858	500	845	604	398	208
1015	751	427	647	455	300	59
924	665	393	672	470	310	221
900	627	415	519	365	238	0
852	610	372	592	422	277	16
815	601	343	395	277	191	97
725	507	337	439	327	197	99
724	520	316	528	394	222	258
708	531	300	487	367	209	0
708	520	310	408	300	187	0
690	490	310	545	405	225	49
677	501	277	360	259	152	39
673	490	291	441	332	183	287
651	482	271	432	295	211	100
645	481	274	472	339	228	29
638	456	285	450	319	208	38
636	459	283	456	321	232	39
631	455	274	402	290	180	27
628	462	269	421	293	203	52
622	419	296	406	295	178	35
612	444	267	415	310	183	54
612	444	259	326	251	142	86
607	446	259	394	285	177	92
600	455	248	466	334	218	16
591	430	262	444	315	203	124
583	428	247	381	279	168	0
582	424	256	378	276	165	97
576	404	256	470	324	231	0
575	423	247	353	244	169	0
565	419	227	459	332	206	236
544	392	242	299	214	140	25
543	392	246	444	323	203	33
543	405	233	344	246	157	196
538	405	231	412	300	186	0
536	393	233	356	261	175	143
524	372	234	329	229	158	88
515	373	221	357	265	145	32
515	377	215	350	247	170	141
470	359	190	363	263	169	55
455	327	197	437	317	192	0
448	343	163	224	167	99	33
435	314	196	328	241	150	36
426	315	185	263	191	115	0
424	312	169	342	271	135	36
418	307	178	337	240	154	76
412	301	179	253	187	119	22
412	305	172	182	139	75	17
404	295	178	369	255	160	66
401	295	165	263	199	105	0
399	315	151	234	168	106	44
399	288	172	245	176	118	93
396	267	187	287	206	130	42
392	281	166	239	181	97	0
390	273	173	219	152	105	0
389	287	163	354	242	167	42
387	282	163	319	240	147	93
380	285	156	277	210	116	44
379	269	175	316	218	152	25
363	271	152	251	193	107	33
363	262	157	278	194	124	152
362	256	163	252	193	105	38
358	253	162	250	183	121	21
357	254	154	159	110	76	26
356	258	157	213	153	99	91
355	258	152	211	145	105	108
354	251	157	222	162	104	34
350	246	155	371	270	162	21
344	255	146	278	199	135	76
339	237	161	167	121	75	54
338	254	135	274	184	141	21
335	242	132	287	212	128	62
333	245	137	256	183	121	44
333	248	131	236	168	110	22
331	242	142	221	141	125	68
323	242	130	251	175	123	0
321	230	150	318	231	142	147
320	244	135	263	174	134	0
317	230	139	214	149	107	29
316	244	123	273	195	131	67
316	228	140	256	187	118	74
312	231	127	226	169	94	49
302	234	119	348	261	140	20
299	219	132	200	145	87	0
299	220	129	175	127	73	23
294	213	127	225	166	101	0
292	216	125	215	154	102	19
286	201	127	226	153	113	70
285	216	121	244	173	111	79
282	185	141	135	105	53	0
280	208	121	195	128	97	24

(continued)

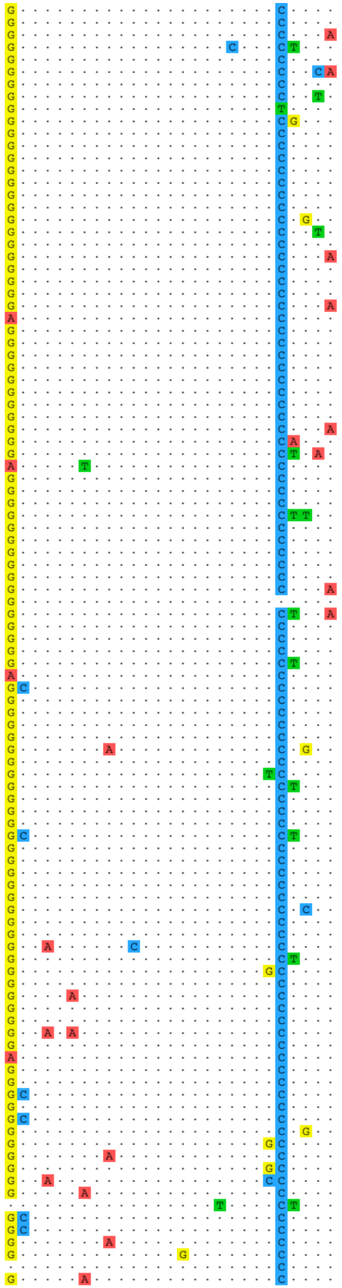

279	205	119	185	136	86	53
278	197	117	221	148	112	0
274	198	116	177	128	83	18
271	192	124	181	125	84	0
270	201	107	85	65	40	13
267	189	125	125	97	52	93
262	180	119	139	104	65	0
260	182	118	95	65	42	28
260	197	105	103	78	47	22
258	200	102	160	113	78	58
257	192	103	177	127	78	0
250	191	95	131	100	53	14
246	174	118	197	144	87	44
243	179	104	172	120	84	30
238	182	91	190	128	92	64
233	174	91	120	89	45	11
224	178	85	210	152	96	17
223	156	107	144	102	67	0
222	171	87	77	60	27	16
218	158	101	139	93	70	0
216	163	85	179	130	77	26
216	143	106	135	93	74	0
214	162	85	145	114	62	0
214	140	99	174	131	72	0
213	160	88	150	102	70	0
210	146	95	163	116	79	0
207	149	88	127	94	60	38
205	154	82	128	99	59	0
202	145	87	143	102	68	49
199	145	89	113	82	52	0
197	143	83	145	108	70	34
195	145	80	138	99	56	26
192	144	81	116	82	47	0
191	144	76	156	102	81	23
191	142	85	181	141	67	37
186	125	85	117	78	57	0
183	134	75	122	94	50	0
183	138	67	119	92	50	0
177	133	78	109	73	55	25
174	130	70	109	75	59	0
173	124	78	108	83	48	0
171	124	75	157	117	64	0
168	121	71	108	82	45	0
167	120	70	179	135	81	0
152	108	67	92	61	44	42
149	108	62	104	72	46	0
149	108	69	161	125	68	0
147	110	61	140	104	51	0
147	100	59	120	82	55	16
145	101	68	87	51	43	0
137	106	47	127	96	51	0
132	94	56	60	39	28	0
132	101	53	122	94	55	17
130	102	51	92	68	37	44
128	98	40	128	93	60	69
128	95	53	128	85	65	0
127	97	55	80	55	43	0
126	89	60	103	73	42	73
125	97	52	39	21	12	0
125	90	64	110	79	54	0
116	90	46	61	43	20	0
116	82	52	145	103	66	0
112	84	49	89	62	41	0
111	65	54	77	51	37	0
110	84	46	120	88	53	42
107	84	43	76	54	36	218
100	77	35	60	39	29	0
95	69	39	98	69	45	16
90	68	34	79	60	32	0
89	63	41	92	66	34	0
88	62	34	110	76	54	0
87	64	36	150	108	72	0
87	57	39	73	51	33	0
85	74	18	56	46	14	0
83	59	38	87	65	37	53
81	67	35	129	101	51	0
73	60	26	43	30	0	0
73	54	33	62	44	28	0
72	55	26	51	36	26	0
70	52	24	37	28	0	376
69	47	31	0	0	0	0
69	53	28	38	21	0	0
67	50	24	29	23	0	0
63	47	22	51	0	24	0
62	48	31	85	63	38	15
61	52	12	108	69	49	0
59	42	28	53	39	0	0
58	47	15	23	0	0	0
57	38	29	22	16	0	0
55	41	20	29	16	0	0
47	27	26	44	24	24	0
41	29	16	64	44	33	0
36	26	20	0	0	0	0
32	19	18	0	0	0	0
25	18	0	0	0	0	0
24	18	11	14	0	0	0
18	14	0	0	0	0	0
15	13	0	0	0	0	0
12	0	0	0	0	0	0
0	0	0	15	0	0	0
0	0	0	14	0	0	0
0	0	0	14	11	0	0
0	0	0	0	0	0	272
0	0	0	12	0	0	0

Supplementary Figure 3. Full list of the off-target sites for *As*Cpf1-Site 6.

Supplementary Figure 3. Full list of off-target sites for *As*Cpf1-Site 6. Tag-seq detected the genome-wide specificity of *As*Cpf1 at Site 6 in HEK293T and MCF7 by the cleanup and all-in-one methods with different numbers of sequencing raw reads (1Gb, 2Gb and 3Gb). The target sgRNA was shown in the first line and the on-target and off-target sites were shown without or with mismatches to the sgRNA sequence by color highlighting. Sequencing read counts and raw data amounts were shown to the right.

Supplementary Figure 4

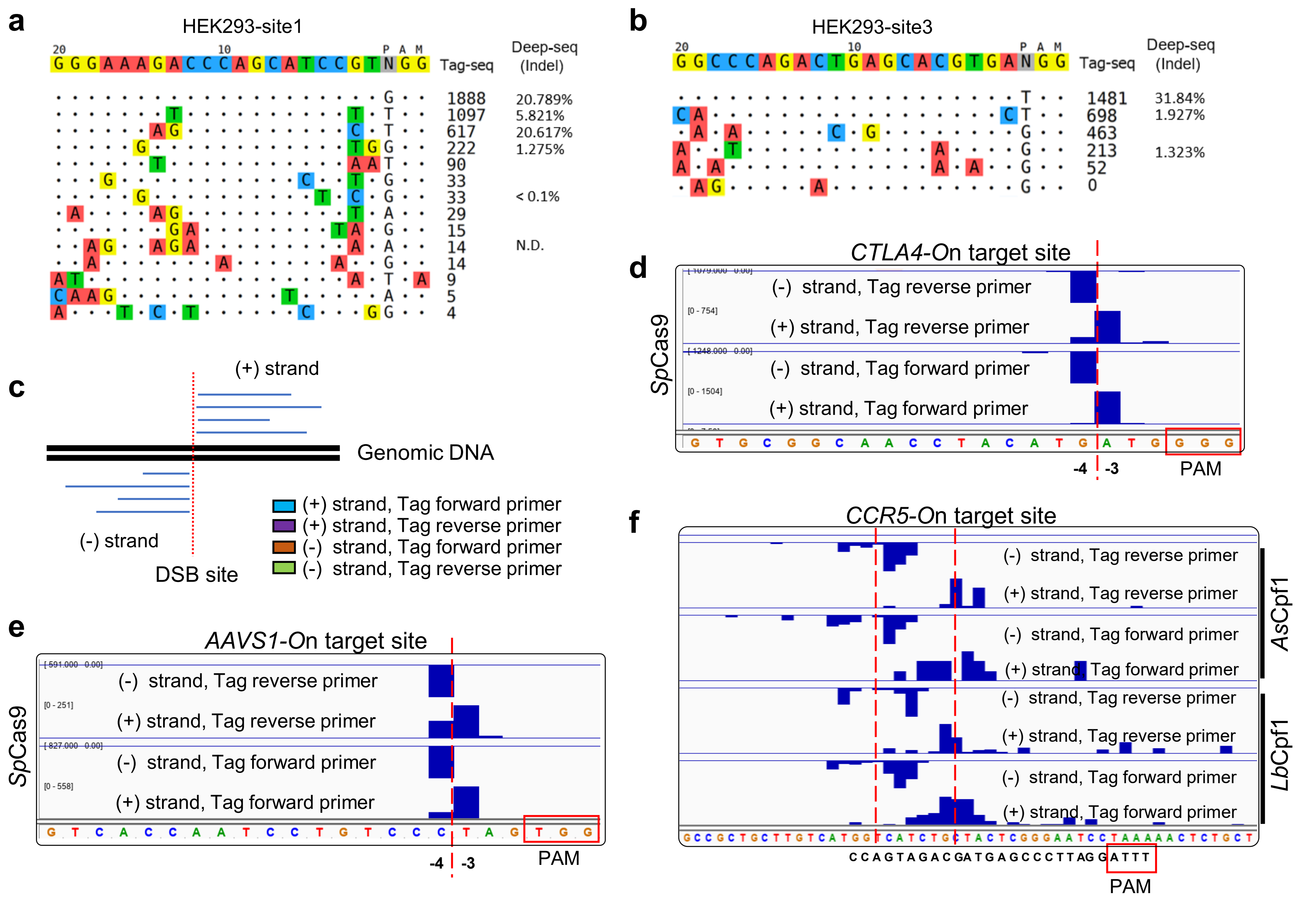

Supplementary Figure 4. Global profiling and molecular characterization of Cas-induced DSBs with Tag-seq.

Supplementary Figure 4. Global profiling and molecular characterization of Cas-induced DSBs with Tag-seq. (a, b) Tag-seq examined the off-target cleavages of HEK293-site1 and HEK293-site3 loci induced by *Sp*Cas9 in HEK293T cell, some of the sites were verified by deep-seq at the left channel. N.D., no detection. **(c)** Diagram of the distribution of the genomic sequence reads identified by Tag-seq libraries. **(d, e)** Tag-seq displayed the characteristics of the *Sp*Cas9-induced-DSBs. Cutting at 3-4 bp upstream from the NGG PAM site. **(f)** Tag-seq displayed the characteristics of the Cpf1-induced-DSBs. Resulting multiple overhangs at *CCR5* in HEK293T or MCF7 cells. Red dotted lines indicate cutting sites. PAM, protospacer adjacent motif.

Supplementary Figure 5

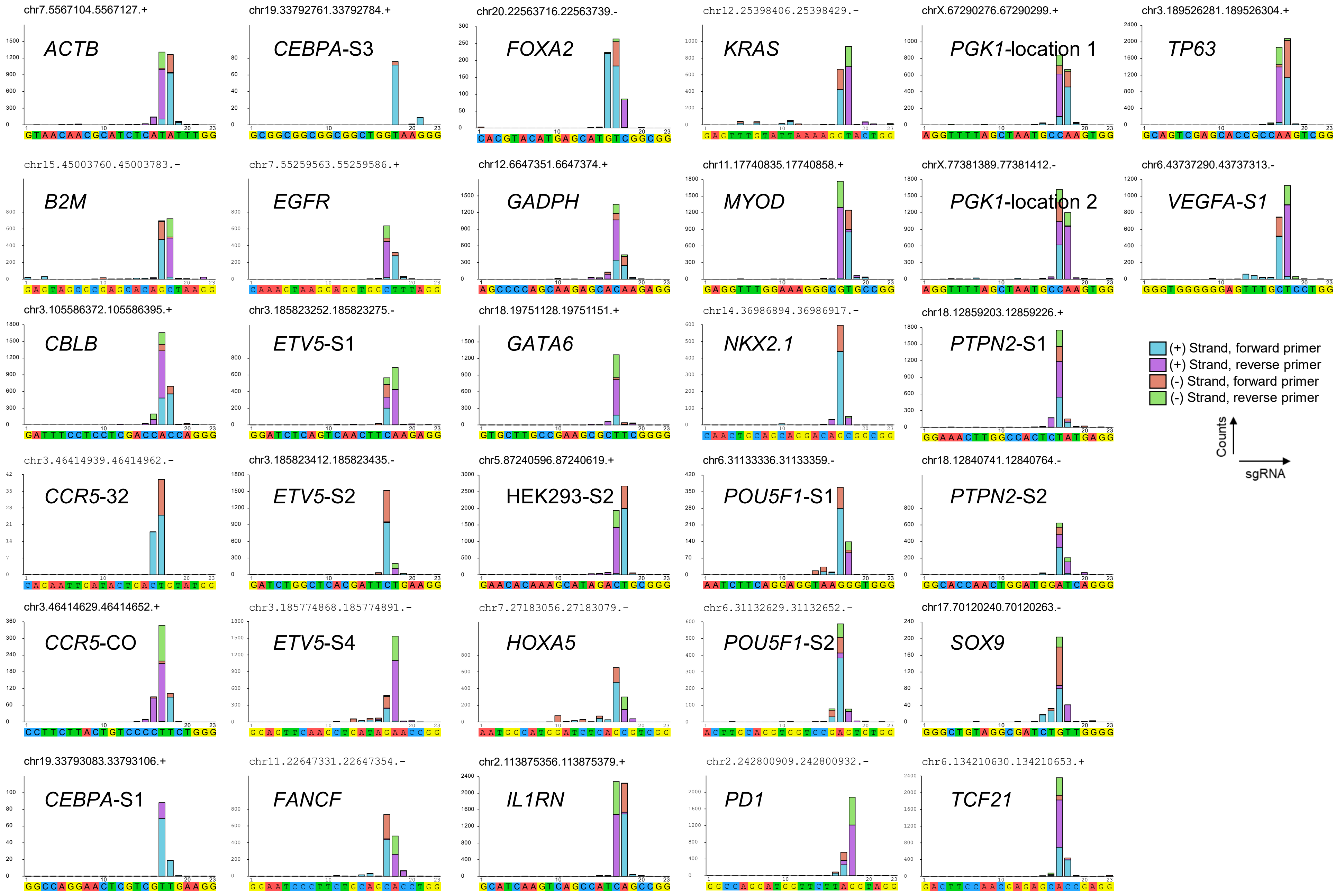

Supplementary Figure 5. Distributions of characteristics of the Tag-oligo integration at target sites induced by *Sp*Cas9.

Supplementary Figure 5. Distributions of characteristics of the Tag-oligo integration at target sites induced by *Sp*Cas9. Sequencing reads were mapped back to the reference (Human hg19) for visualization of the localization of the DSBs. Mapped reads for the on-target sites of the 31 sites for *Sp*Cas9 assessed parallelly by Tag-seq are shown. The target sequences were shown with the 20-bp spacer sequence upstream from the NGG PAM site on the *x* axis, and the peaks were located 3-4 bp upstream from the NGG PAM, which were the expected positions of the *Sp*Cas9 cleavage events.

Supplementary Figure 6

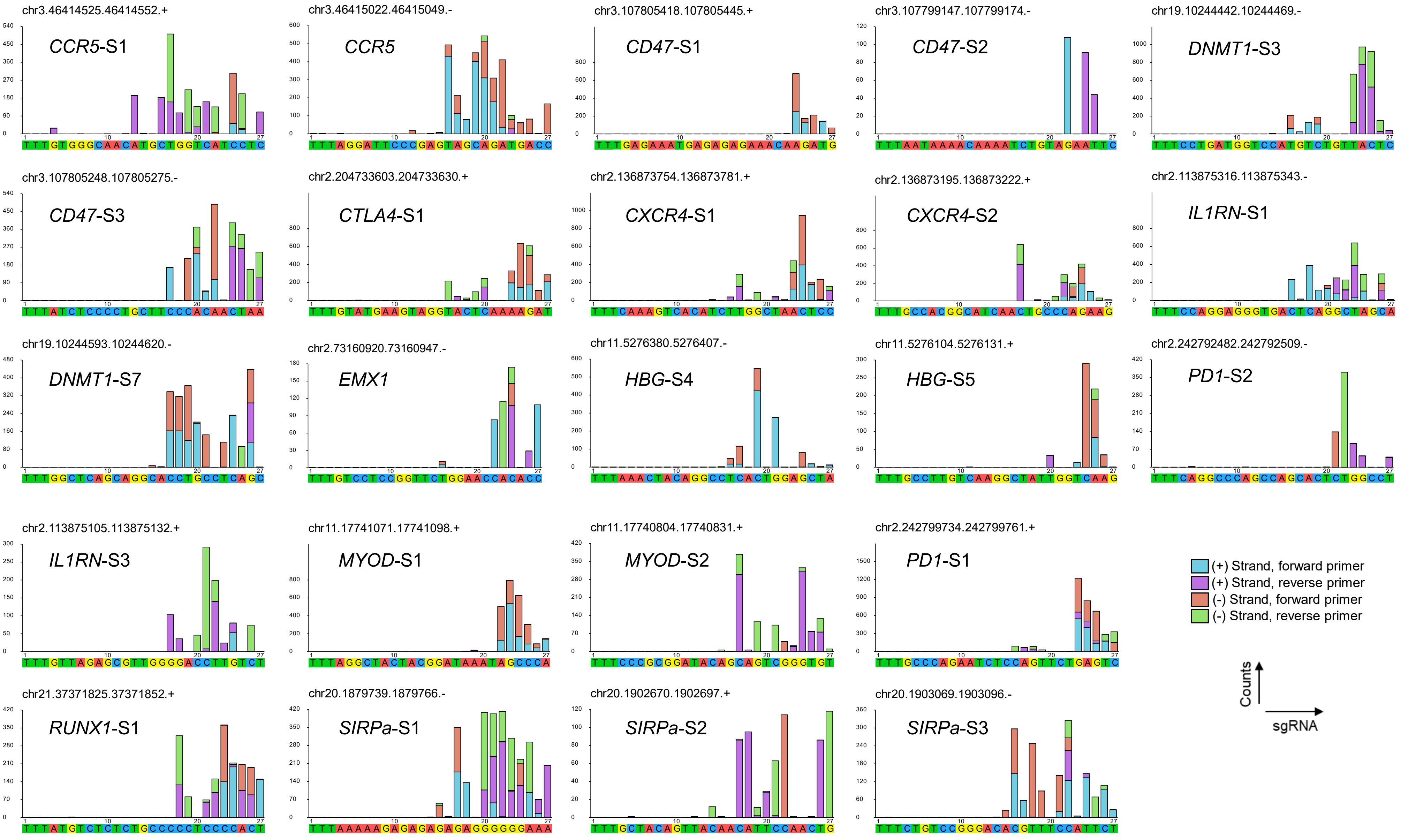

Supplementary Figure 6. Distributions of characteristics of the Tag-oligo integration at target sites induced by *As*Cpf1.

Supplementary Figure 6. Distributions of characteristics of the Tag-oligo integration at target sites induced by *As*Cpf1. Sequencing reads were mapped back to the reference (Human hg19) for visualization of the localization of the DSBs. Mapped reads for the on-target sites of the 23 sites for *As*Cpf1 assessed parallelly by Tag-seq are shown. The target sequences were shown with the 23-bp spacer sequence downstream from the TTTN PAM site on the *x* axis, and the peaks displayed multiple overhangs, which were the expected positions of the Cpf1 cleavage events.

Supplementary Figure 7

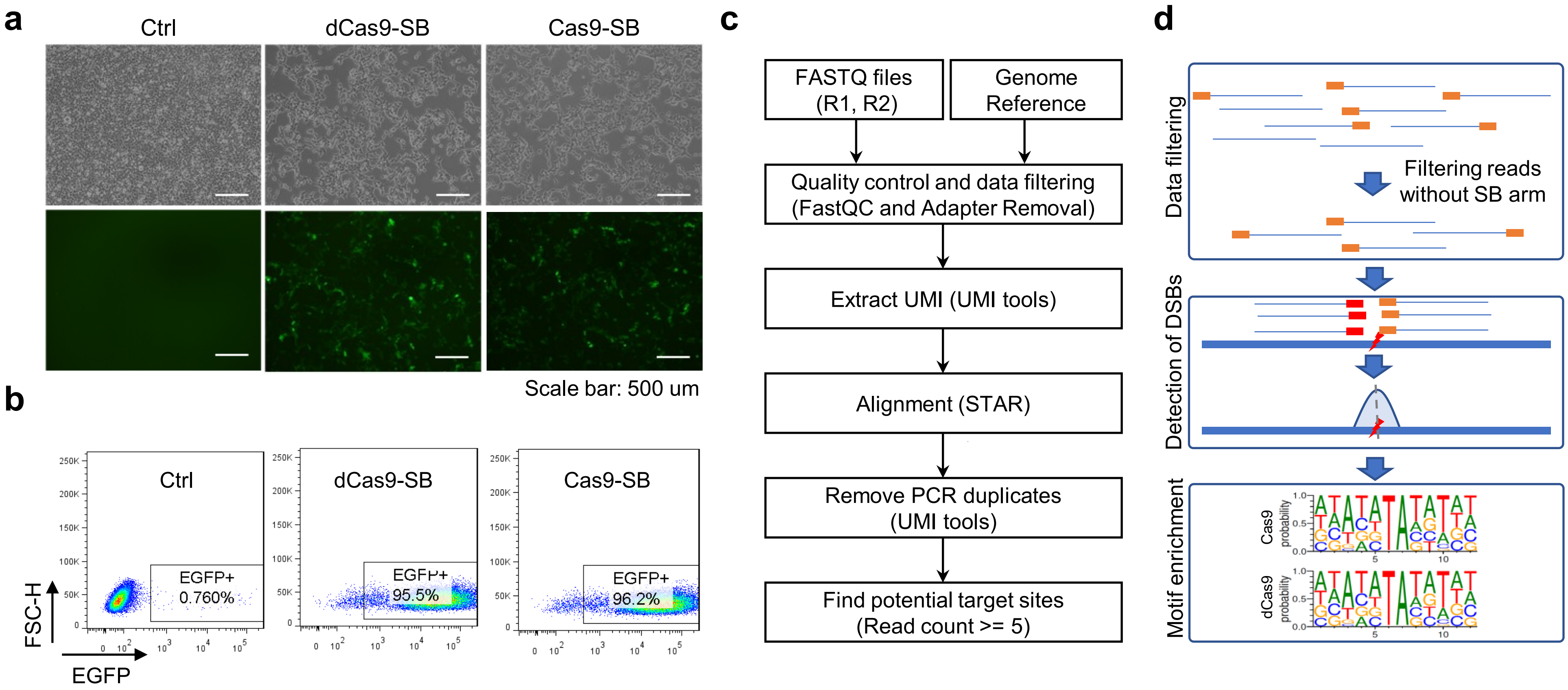

Supplementary Figure 7. Tag-seq discovers integration sites of EGFP induced by transposons.

Supplementary Figure 7. Tag-seq discovers integration sites of EGFP induced by transposons. (a, b) Microscopy and FACS analyses of EGFP expression in the dCas9-SB and the Cas9-SB transfected cells. Ctrl: un-transfected HEK293T cell. Cells were harvested 21 days after a three-day puromycin selection starting at day 2 after transfection. **(c)** Schematic of Tag-seq detection of the integrated locations induced by Sleeping Beauty transposons. **(d)** Tag-seq data-analysis scheme for Cas9-SB and dCas9-SB, containing three parts: data filtering, detection of DSBs and motif enrichment.

Supplementary Table 1. Procedures, times and costs comparison between GUIDE-seq and Tag-seq.

Content	GUIDE-seq		Tag-seq	
	Steps and times	Cost	Steps and times	Cost
Transfection	✓ (2-3 days)	Lonza Kit<br>~ 25 USD/test	✓ (2-3 days)	PEI<br>less 1 USD/test
Genomic DNA preparation	✓ (20 min)	~ 1.5 USD/test	✓ (20 min)	~ 1.5 USD/test
Genomic DNA fragmentation	✓ (sonication and then cleanup, 60 min)<br>(required extra steps)	~ 15 USD/test	✓ (combination in a single-tube reaction, 50 min)	~ 15 USD/test
End repair, A-tailing and Ligation	✓ (105 min)	~ 15 USD/test		
PCR	✓ (150 min)<br>✓ (1st PCR cleanup, 30min)<br>(required extra steps)	~ 8 USD/test<br>~ 8 USD/test	✓ (all-in-one PCR, 150 min)	~ 8 USD/test
Sequencing	✓<br>✓ (Output Index2)<br>(required extra steps)	~ 24-32* USD/libaray	✓	~ 14-21* USD/libaray
Total	Steps: 8<br>Times:<br>2-3d + 365 min	~ 96.5-104.5 USD/test	Steps: 5<br>Times:<br>2-3d + 220 min	~ 39.5-46.5 USD/test

Note: "✓"means requiring a procedure. " * ", GUIDE-seq usually sequences a library with a average depth of 300-400 Mb in Miseq device[1], and Miseq can produce output data of up to 15 Gb[2] and its per run cost in China is about 1200 USD. Thus, each library for GUIDE-seq in Miseq platform will approximately spend 24-32 USD. While we generally sequence Tag-seq with a depth of 2-3 Gb raw data in Hiseq/Novaseq, because this depth leads to the comparable counts detected by GUIDE-seq, and this depth in China will spend about 14-21 USD.

References:

1. Tsai SQ, *et al.* GUIDE-seq enables genome-wide profiling of off-target cleavage by CRISPR-Cas nucleases. *Nat Biotechnol* **33**, 187-197 (2015).

2. Ravi RK, Walton K, Khosroheidari M. MiSeq: A Next Generation Sequencing Platform for Genomic Analysis. *Methods Mol Biol* **1706**, 223-232 (2018).

Supplementary Table 2. Detailed comparison of GUIDE-seq, GUIDE-seq-based methods.

Contents \ Methods	GUIDE-seq	iGUIDE	TTISS	Tag-seq
Oligo<br>GC%	dsODN (34 bp)<br>26.5%	i-dsODN (46 bp)<br>39.1%	dsDonor (47 bp)<br>57.4%	Tag (35 bp)<br>45.7%
UMI and SB positions in adapter	Before Read 1 Primer	Before Read 1 Primer	Unknown	After Read 1 Primer
Transfection Reagent	Lonza Nucleofection	Lonza Nucleofection	GeneJuice	Polyetherimide (PEI) (Routine reagents)
Fragmentation, end repair, dA-tailing, and adapter ligation	Separated sequential steps	Separated sequential steps	Combined in Tn5 fragmentation	Combined in a single-tube reaction
First PCR Cleanup	Yes	No	No	No (all-in-one)
DSBs analysis required files	R1: gRNA<br>R2: dsODN+gDNA<br>I1: P7 index<br>I2: SB+UMI	R1: gRNA<br>R2: dsODN+gDNA<br>I1: P7 index<br>I2: SB+UMI	Unknown	R1: UMI+SB+gDNA<br>R2: Tag+gDNA<br>I1: P7 index
Users	A few of labs<br>(very suitable for the private sequencer)	A few of labs<br>(very suitable for the private sequencer)	Most labs<br>(rely on TN5 preparation)	Nearly all labs<br>(perfectly fits the sequencing vendor)

Supplementary Table 3. The donor DNA used in this study.

Name	Sequence (5′–3′)	Note
Oligo-1-F	P-A*T*AATATGTGTATTATAATAAAAGAAATATTAG*T*T	35bp, GC=11%
Oligo-1-R	P-A*A*CTAATATTTCTTTTATTATAATACACATATT*A*T	35bp, GC=11%
Tag-F	P-A*T*CTCTGAGCCTTATGCGAAATGCGTGTTATCG*C*A	35bp, GC=46.7%
Tag-R	P-T*G*CGATAACACGCATTTCGCATAAGGCTCAGAG*A*T	35bp, GC=46.7%
Oligo-2-F	P-G*G*AAGGGCTCGAGCCTGCCCAACTCGCACCGCG*G*G	35bp, GC=74%
Oligo-2-F	P-C*C*CGCGGTGCGAGTTGGGCAGGCTCGAGCCCTT*C*C	35bp, GC=74%

Note: P, phosphorylation; *, phosphorothioate linkage.

Supplementary Table 4. Primers for Deep-seq.

Primers	Sequence (5′–3′)
RUNX1-DS-F-Cpf1	atcgCTCTTTCCCTACACGACGCTCTTCCGATCTAGAGTAGAGCCATCGCTTCCTCCTGAAA
RUNX1-DS-R-Cpf1	ctctACTGGAGTTCAGACGTGTGCTCTTCCGATCTGCTCAGGAAGTCCCAGAGGTATCCAGCA
SITE6-DS-F-Cpf1	atcgCTCTTTCCCTACACGACGCTCTTCCGATCTGACATTGTCCACATCCTCACCACCTGTTT
SITE6-DS-R-Cpf1	ctctACTGGAGTTCAGACGTGTGCTCTTCCGATCTAGCGTAGCGGACAGGGTGGGTTACCTTG
CCR5-DS-F-Cpf1	atcgCTCTTTCCCTACACGACGCTCTTCCGATCTACTGCATAGTTTGCGTCTCTCCCAGGAA
CCR5-DS-R-Cpf1	ctctACTGGAGTTCAGACGTGTGCTCTTCCGATCTCATGCCTACCCTGTGCCTCTTCTTCTCA
DNMT1-DS-F-Cpf1	atcgCTCTTTCCCTACACGACGCTCTTCCGATCTTAGATCGCGTCACTCTGGGGAACACGC
DNMT1-DS-R-Cpf1	atcgCTCTTTCCCTACACGACGCTCTTCCGATCTCTCTCTATGTCACTCTGGGGAACACGC
AAVS1-DS-F-Cas9	gCTCTTTCCCTACACGACGCTCTTCCGATCTACTGCAGGTGGGGGTTAGACCCAATATC
AAVS1-DS-R-Cas9	ACTGGAGTTCAGACGTGTGCTCTTCCGATCTCATGCCGGTTAATGTGGCTCTGGTTCTG

Supplementary Methods

The detailed protocol of Tag-seq

1. Equipment:

- Tabletop centrifuge (Eppendorf)
- Nanodrop 2000 (Thermo Scientific)
- 12-Tube Magnetic Separation Rack (NEB)
- Thermocycler (Thermo Scientific)
- HiSeq/NovaSeq sequencing platform (Commercial)

2. Materials:

- Polyetherimide (PEI), (Polysicence Inc, 61128-46-9)
- TIANamp Genomic DNA Kit (TIANGEN-China, DP304)
- Fragmentation, End Preparation & dA-tailing module and Adapter Ligation Module (Vazyme-China, ND617)
- Hieff NGS™ DNA Selection Beads (YEASEN-China,12601ES08)
- KOD-Plus: (TOYOBO, KOD-201)
- Nuclease free water
- 1x TE (10mM Tris, 1mM EDTA, pH 8.0)
- 200-proof (100%) Ethanol, (Sigma Aldrich)
- TetraMethylAmmonium Chloride (TMAC) 5M, (Sigma Aldrich)

Protocol for Tag-seq Library Preparation

1. Oligo Preparation:

➢ Tag sequence Preparation:

The Tag DNA is made by annealing the Tag-F with the Tag-R.

1X TE Buffer	80.0 µL
Tag-F (100µM)	10.0 µL
Tag-R (100µM)	10.0 µL
**Total**	**100.0 µL**

Annealing program: 95°C for 5min; slow ramp down (approximately -0.1°C/sec) to 4°C; hold at 4°C. Store in -20°C.

- Y-adapter Preparation：

The improved Y-adapters (UMI and sample barcode after Read 1 Primer) are made by annealing the adapter-X (X=1-12, Supplementary Table 4) oligo with the common adapter-R.

1X TE Buffer	80.0 μL
Adapter-X (100μM)	10.0 μL
Common adapter-R (100μM)	10.0 μL
**Total**	**100.0 μL**

Annealing program: 95°C for 1 s; 60°C for 1s; slow ramp down (approximately -0.1°C /sec) to 4°C; hold at 4°C. Store in -20°C.

2. Library template gDNA Preparation:

- Cell transfection: HEK293T/MCF7 cells grow up to nearly 70% per well in 24-well plate, then were transfected by PEI reagent using the following scheme:

	Reagents	Dose	Description
Solution A	PEI	2 μg/ml	
	Free DMEM	50 μl	
			Mix and stand at RT, 5 min
Solution B	Cas9/Cpf1	250 ng	
	sgRNA	250 ng	
	Tag	10 nM	
	Free DMEM	50 μl	Mix and stand at RT, 5 min

Mix Solution A and Solution B at RT for 15~30 min, then added to the cell media.

- Genomic DNA (gDNA) extraction: Approximately 48-72 h post-transfection, cells were collected and gDNA was extracted using TIANamp Genomic DNA Kit (TIANGEN, China) accordance to the manufacturer’s instructions. gDNA were finally diluted in TE Buffer and store at -20°C.

Approximately 800 ng gDNA were used for library (L) and library (R) preparation.

- Notice: The following step should be performed on ice.

- gDNA Fragmentation, End repair & A-tailing (One steps):

DNA	800.0 ng
FEA buffer	2.5 μL
ddH_2O up to	17.5 μL
FEA Enzyme Mix	5.0 μL
**Total**	**25.0 μL**

Fragmentation, End repair & A-tailing Thermocycler Program: 37°C for 5min, 65°C for 30min; hold at 4°C.

- Adapter ligation (directly add):

DNA (from previous step)	25.0 μL
Rapid ligation buffer 3	12.5 μL
Annealed Adapter-X (10 μM)	2.0 μL
Rapid DNA Ligase	2.5 μL
ddH_2O up to	8.0 μL
**Total**	**50.0 μL**

Adapter ligation Thermocycler Program: 20°C for 15 min; hold at 4°C.

DNA Cleanup: 0.8X DNA Selection Beads (YEASEN, 40 μl), elute in 20 μL TE buffer. For Library (L) and Library (R) construction, use 10 μL DNA template for each library.

3. Library construction (All-in-one):

1st PCR: For Library (L) and Library (R) preparation, make separate PCR reactions using the Tag-L-F1 or the Tag-R-F1 primer, respectively.

ddH2O	10.7 μL
10X Buffer for KOD Polymerase	3.0 μL
dNTP mix, 10mM	0.6 μL
MgSO4, 25 mM	2.4 μL
KOD polymerase, 1 U/μl	0.3 μL
Tag-L-F1/Tag-R-F1 Primer (10μM)	1.0 μL
TMAC (0.5M)	1.5 μL
P5, 10 μM	0.5 μL
DNA (from previous step)	10.0 μL
**Total**	**30.0 μL**

1st PCR Thermocycler Program (30μL, touchdown):

94°C for 5 min,

14 cycles of [94°C 30 s, 68°C (-1°C/cycle) 2 min, 68°C 30 s],

9 cycles of [94°C 30 s, 55°C 1 min, 68°C 30 s],

68°C 5 min, 4°C hold.

2nd PCR: For Library (L) and Library (R) construction, make separate PCR reactions using the Tag-L-F2 or the Tag-R-F2 primer, respectively.

10X Buffer for KOD Polymerase	2.0 μL
dNTP mix, 10mM	0.4 μL
$MgSO_4$, 25 mM	1.6 μL
KOD polymerase, 1 U/μl	0.2 μL
Tag-L-F2/Tag-R-F2 Primer (10μM)	1.0 μL
TMAC (0.5M)	1.5 μL
P5, 10 μM	0.5 μL
P7-X（X=1, 2, 3, 4…）	1.5 μL
ddH_2O	11.3 μL

2nd PCR master Mix (Total)	20.0 μL
1st PCR product	30.0 μL
**Total**	**50.0 μL**

2nd PCR Thermocycler Program (50μL, touchdown):

94°C for 5 min,

14 cycles of [94°C 30 s, 68°C (-1°C/cycle) 2 min, 68°C 30 s],

9 cycles of [94°C 30 s, 55°C 1 min, 68°C 30 s],

68°C 5 min, 4°C hold.

DNA Cleanup: 0.7X DNA Selection Beads (YEASEN, 35 μL), elute in 20 μL TE buffer. Store at -20°C.

Library Sequencing

1. Library samples are sequenced commercially by paired-end read sequencing using the Illumina Miseq/Hiseq/Novaseq platform according to the manufacturer instruction.

2. Sequencing data (including R1 and R2) in a fastq format are used for downstream bioinformatics analysis using the Tag-seq pipeline (https://github.com/zhoujj2013/Tag-seq or https://doi.org/10.5281/zenodo.4679460.). L and R libraries are needed for each test.
