# Supplementary material for: Tag-seq: a convenient and scalable method for genome-wide specificity assessment of CRISPR/Cas nucleases
Source: Commun Biol. 2021 Jul 2;4:830. doi: 10.1038/s42003-021-02351-3 (PMC8253812; doi:10.1038/s42003-021-02351-3)
Supplement: Supplementary file 3 — Description of Supplementary Files [file 42003_2021_2351_MOESM3_ESM.pdf]

Description of Supplementary Data Files

File name: Supplementary Data 1-4
Description:

Supplementary Data 1. *Comparison of the methods for mapping DNA double-strand breaks.* The existed methods for genome-wide identification of potential Cas-nuclease-induced double-strand breaks (DSBs) can be generally divided into two categories, cell-free and cell-based methods, which were listed and compared in terms of improvement, main features, advantages, and disadvantages.

Supplementary Data 2. *sgRNAs used in this study.* The sgRNAs (including both *Sp*Cas9 and Cpf1) and their location in genome were listed in this table.

Supplementary Data 3. *Primers and oligos for Tag-seq.* The donor DNA sequence Tag-oligo and the PCR primers that used in Tag-seq method were listed in this table.

Supplementary Data 4. *Source data values for Supplementary Fig. 1d, e and Supplementary Fig. 5, 6.* For the source data values of Supplementary Fig. 1d, the integration rate was calculated as the ratio of the Gray levels of the PCR produce using Tag-primer and R primers to the Gray levels of the PCR produce using gene specific F and R primers, please see Supplementary Fig. 2 for more details. For the source data values of Supplementary Fig. 1d, the integration rate was calculated as the ratio of read counts with donor DNA sequence to the total sequencing read counts. For the source data values of Supplementary Fig. 5, 6, the values is the location of the Tag insertion into the genome, which were generated by the Tag-seq pipeline, which was available at https://github.com/zhoujj2013/Tag-seq and https://doi.org/10.5281/zenodo.4679460. Sequencing data for Supplementary Fig. 1e and Supplementary Fig. 5, 6 can be found in NCBI (Bioproject PRJNA678456).
