# Supplementary material for: Tag-seq: a convenient and scalable method for genome-wide specificity assessment of CRISPR/Cas nucleases
Source: Commun Biol. 2021 Jul 2;4:830. doi: 10.1038/s42003-021-02351-3 (PMC8253812; doi:10.1038/s42003-021-02351-3)
Supplement: Supplementary file 5 — Reporting Summary [file 42003_2021_2351_MOESM5_ESM.pdf]

nature research

Corresponding author(s): Zhili Rong
Last updated by author(s): May 28, 2021

Reporting Summary

Nature Research wishes to improve the reproducibility of the work that we publish. This form provides structure for consistency and transparency in reporting. For further information on Nature Research policies, see our Editorial Policies and the Editorial Policy Checklist.

Statistics

For all statistical analyses, confirm that the following items are present in the figure legend, table legend, main text, or Methods section.

n/a	Confirmed	
☐	☒	The exact sample size (n) for each experimental group/condition, given as a discrete number and unit of measurement
☐	☒	A statement on whether measurements were taken from distinct samples or whether the same sample was measured repeatedly
☐	☒	The statistical test(s) used AND whether they are one- or two-sided<br>*Only common tests should be described solely by name; describe more complex techniques in the Methods section.*
☒	☐	A description of all covariates tested
☒	☐	A description of any assumptions or corrections, such as tests of normality and adjustment for multiple comparisons
☒	☐	A full description of the statistical parameters including central tendency (e.g. means) or other basic estimates (e.g. regression coefficient) AND variation (e.g. standard deviation) or associated estimates of uncertainty (e.g. confidence intervals)
☒	☐	For null hypothesis testing, the test statistic (e.g. F, t, r) with confidence intervals, effect sizes, degrees of freedom and P value noted<br>*Give P values as exact values whenever suitable.*
☒	☐	For Bayesian analysis, information on the choice of priors and Markov chain Monte Carlo settings
☒	☐	For hierarchical and complex designs, identification of the appropriate level for tests and full reporting of outcomes
☐	☒	Estimates of effect sizes (e.g. Cohen's d, Pearson's r), indicating how they were calculated

Our web collection on statistics for biologists contains articles on many of the points above.

Software and code

Policy information about availability of computer code

Data collection	Next-generation sequencing data(Tag-seq and Deep-seq) were collected with commercial Illumina Novaseq/Hiseq instruments. FACS data were generated using a BD FACSAria II.
Data analysis	All code for Tag-seq analysis pipeline in this study is available at https://github.com/zhoujj2013/Tag-seq and https://doi.org/10.5281/zenodo.4679460. Any updates will also be published on Zenodo and GitHub. Deep-seq data were analyzed with the online methods in this study, FACS Data were analyzed with the FlowJo software V7 (TreeStar, USA).

For manuscripts utilizing custom algorithms or software that are central to the research but not yet described in published literature, software must be made available to editors and reviewers. We strongly encourage code deposition in a community repository (e.g. GitHub). See the Nature Research guidelines for submitting code & software for further information.

Data

Policy information about availability of data

All manuscripts must include a data availability statement. This statement should provide the following information, where applicable:

- Accession codes, unique identifiers, or web links for publicly available datasets
- A list of figures that have associated raw data
- A description of any restrictions on data availability

All the sequencing data related to this study have been deposited in NCBI (Bioproject PRJNA678456). And other data that support the findings of this study are available from the corresponding author upon reasonable request. Beside, the source data for Supplementary Figure 1d-e, and 5, 6. are shown in Supplementary Data 4.

Field-specific reporting

Please select the one below that is the best fit for your research. If you are not sure, read the appropriate sections before making your selection.

☒ Life sciences ☐ Behavioural & social sciences ☐ Ecological, evolutionary & environmental sciences

For a reference copy of the document with all sections, see nature.com/documents/nr-reporting-summary-flat.pdf

Life sciences study design

All studies must disclose on these points even when the disclosure is negative.

Sample size	Sample sizes were determined based on other researches that generate reproducible results in similar experimental contexts.
Data exclusions	No data were excluded.
Replication	Independent replicates were performed with different test.
Randomization	No samples were randomized. Controls were run in parallel whenever applicable to control for covariates.
Blinding	Blinding was not performed.

Reporting for specific materials, systems and methods

We require information from authors about some types of materials, experimental systems and methods used in many studies. Here, indicate whether each material, system or method listed is relevant to your study. If you are not sure if a list item applies to your research, read the appropriate section before selecting a response.

Materials & experimental systems

n/a	Involved in the study	
☒	☐	Antibodies
☐	☒	Eukaryotic cell lines
☒	☐	Palaeontology and archaeology
☒	☐	Animals and other organisms
☒	☐	Human research participants
☒	☐	Clinical data
☒	☐	Dual use research of concern

Methods

n/a	Involved in the study	
☒	☐	ChIP-seq
☐	☒	Flow cytometry
☒	☐	MRI-based neuroimaging

Eukaryotic cell lines

Policy information about cell lines

Cell line source(s)	HEK293T cells (ATCC), MCF7 cells (ATCC).
Authentication	Cell line identities were confirmed by STR profiling (ATCC).
Mycoplasma contamination	Supernatant was analyzed for the presence of Mycoplasma and every test was negative.
Commonly misidentified lines (See ICLAC register)	No commonly misidentified cell lines were used in this work.

Flow Cytometry

Plots

Confirm that:

☒ The axis labels state the marker and fluorochrome used (e.g. CD4-FITC).

☒ The axis scales are clearly visible. Include numbers along axes only for bottom left plot of group (a 'group' is an analysis of identical markers).

☒ All plots are contour plots with outliers or pseudocolor plots.

☒ A numerical value for number of cells or percentage (with statistics) is provided.

Methodology

Sample preparation	Cell culture, transfection, genomic DNA extraction and Library construction are detailedly described in the online methods.
Instrument	FACSAria (BD Biosciences)
Software	BD FACSDiva Software(BD Biosciences) and FlowJo software (TreeStar, USA).
Cell population abundance	Cell population abundances were performed for testing the EGFP insertion efficiency induced by transposase. HEK293T cells were harvested 21 days for complete degradation of EGFP plasmid after a three-day puromycin selection starting the next day post-transfection. EGFP expressing cells were ~95% GFP-positive.
Gating strategy	Gates were performed by the untransfected control cells.

☒ Tick this box to confirm that a figure exemplifying the gating strategy is provided in the Supplementary Information.
